# Knowledge, attitudes and self-reported practices toward children oral health among mother’s attending maternal and child’s units, Salé, Morocco

**DOI:** 10.1186/s12889-018-5542-2

**Published:** 2018-05-11

**Authors:** Sanaa Chala, Soumia Houzmali, Redouane Abouqal, Faïza Abdallaoui

**Affiliations:** 1 Department of Endodontics and Restorative Dentistry, Faculty of Dental Medicine, BP: 6212, Rabat instituts, Rabat, Morocco; 2 Laboratory of Biostatics, Clinical and Epidemiological Research, Faculty of Medicine and Pharmacy, Mohammed V University in Rabat, Rabat, Morocco; 3 Mohammed V Military Teaching Hospital, Rabat, Morocco; 4 Oral Biology and Biotechnology Laboraty, Mohammed V University in Rabat, Rabat, Morocco

**Keywords:** Knowledge, Attitudes, Practices, Oral health, Mother’s

## Abstract

**Background:**

The occurrence of severe dental caries is particularly prevalent and harmful in children. A better understanding of parental factors that may be indicators of children’s risk of developing dental caries is important for the development of preventive measures.

This study was conducted to assess knowledge, attitudes, and practices (KAP) of mothers in Salé, Morocco regarding oral health and their predictors.

**Methods:**

A cross-sectional KAP study was conducted of Mother and Child units in Salé, Morocco. Mothers attending the selected units from November 2014 to 29 January 2015 were recruited. Data were collected using a semi-structured questionnaire, administered by face-to-face interviews, to record socio-demographic factors and KAPs. The main outcome measures included knowledge about oral health diseases and preventive measures, and attitudes and practices related to oral health prevention measures and dental care. KAPs scores were then recoded based on responses and scores were determined for each KAP domain. Linear regression analysis was conducted to assess predictors of KAP scores.

**Results:**

Among 502 mothers included, 140 (27.8%) were illiterate and 285 (60.9%) were aware that fluoride has a beneficial effect in caries prevention. Mothers’ own practices about dental care were statistically related to their children’s use of dental care services (*p* < 0.001). Multiple linear regression analysis revealed that the knowledge score was associated with mother’s age (β = 0.05; 95% CI; *p* < 0.001), education level, and median income (β = 0.38; *p* = 0.04). Significant predictors of oral health-related practices were mother’s education level and children’s health status.

**Conclusions:**

Limited KAP scores were observed among the studied population. A great emphasis on oral health education and some risk factor modifications are recommended.

**Electronic supplementary material:**

The online version of this article (10.1186/s12889-018-5542-2) contains supplementary material, which is available to authorized users.

## Background

Appropriate infant oral health attitudes and practices are of fundamental importance for preventing chronic oral diseases [1]. Oral health patterns are consolidated during childhood, and some attitudes may increase a child’s risk of caries development [2, 3].

Morbidities due to dental caries are particularly harboured in children from families of low socio-economic level [4], whose nutrition [5, 6] and quality of life may be consequently impaired [3, 7–9]. Potential risk factors of dental caries include biological and behavioural factors, all of which may be modulated by environmental factors [10, 11].

Parents play an important role in promoting positive attitudes and strategies toward oral health behaviours [12, 13]. Mothers are the immediate and reliable caregivers of children in many countries, and they have a central role in providing effective guidance and positive attitudes toward oral health [14, 15].

Despite improvements in oral health measures in high-income countries, the literature notes the persistence of an imbalance in caries prevalence in certain countries [16, 17]. Moreover, most KAP-related surveys concentrate on parents from high-income countries, and less is known from countries with high prevalence of dental caries such as Morocco [17].

Morocco has recently commenced an expanded program on immunisation. Mother and Child units (MCUs) serve as basis of this program, which is administered on a routine and outreach basis. The MCUs in Morocco offer vaccines free of charge to children from birth. They also offer counselling for mothers regarding infant-feeding practices and general health.

The aim of the present study was to assess knowledge, beliefs, attitudes, and practices (KAPs) of mothers attending MCUs regarding infant oral health and preventive measures.

## Methods

### Participants

This study was a cross-sectional survey among mothers attending early childhood services between 12 November 2014 and 29 January 2015, including childcare and mother’s health services in selected MCUs in Salé, in the Kingdom of Morocco. Salé is a city situated on the opposite bank of the Bouregreg River from the capital, Rabat. The total population of Salé in 2014 was 890,403 [18].

The study population was mothers aged 18 years and above who had at least one borne child. Mothers who were less than 18 years old, unable to respond to questions, or declined to participate in the study were excluded.

All mothers who attended the selected units during the period of survey were approached and invited to participate in the survey; this includes either mothers with newborn children attending the units for vaccination or for paediatrics consultation. Participation was strictly voluntary; no subject-identifying data were collected and no incentives were provided.

The institutional review board and the regional health authority approved the study prior to its implementation.

### Data collection

A structured questionnaire to investigate knowledge, attitudes, and practices was developed for the purpose of the study. The questionnaire drew on previous research in this area [13–15, 17, 19, 20], with necessary modifications made considering lifestyle and cultural factors related to the Moroccan population.

The pre-final questionnaire was then pre-tested with 102 mothers. This pre-final form served to test the face validity of the questionnaire and determine how meaningful it was to the studied population. After discussion with participants and interviewer, the variability in responses, understanding of items, and ambiguity were evaluated. The questionnaire was then refined and the final version was used for this study.

The entire survey took place from 12 November 2014 to 29 January 2015, during different periods in each unit. For each unit, the interviewer approached all mothers during the study period to determine eligibility. All eligible mothers were asked if they were interested in participating in the study.

The information was collected by face-to-face interview. The questionnaire was filled out anonymously on site. The interviewer was previously trained to standardise approach to the participants for consent and in application of the questionnaire.

The final version of the questionnaire covered the followings areas (Additional file 1):

- Family socio-economic and demographic variables, including parent’s education level (none, primary, 6 < EL < 12, university), number of children, household income (low, moderate, and high), mother’s employment status (working/non-working), and medical coverage (yes/no). The monthly family income was measured relative to the Moroccan minimum wage during the period of data gathering.
- Mother’s general health rating (good global health/chronic illness), children’s health status (good global health/chronic illness).
- Knowledge was measured using a 10-item questionnaire relating to general knowledge of oral health, including the value of fluoride in dental caries prevention, recommended fluoride dose for children, preventive measures regarding oral bacterial acquisition, relation between primary teeth and permanent teeth, importance of oral health, age to begin teeth brushing, frequency of brushing, nutrition behaviour and dental caries, and importance of preventive dental health. Responses were rated and recoded yes/no/don’t know). The following ranking scale was used to record responses: 1 point per correct answer, 0 for incorrect or don’t know). Scores ranged from 0 to 10, and a higher score indicated a higher level of knowledge.
- Attitudes were measured using a six-item questionnaire. Recorded items included attitudes about oral bacterial acquisition, prevention measures, counselling, and control methods (parental support of infant oral health and nutrition behaviour). The attitudes were first recorded and then classified as positive or negative attitudes.
- Practice domains, including practice patterns regarding oral hygiene measures (frequency and regularity of tooth brushing, fluoridated toothpaste use, sugar consumption, age child commenced tooth brushing, brushing motion), dental attendance (mother’s own dental attendance pattern, child’s dental attendance pattern), and nutritional practices. The practices were first recorded and then classified as positive or negative patterns.

The section regarding attitudes and practices included multiple-choice as well as open-ended questions. The practice patterns section was completed prior to the knowledge and attitudes section to avoid leading answers.

### Data analysis

Characteristics of the study population were presented as median and interquartile ranges for continuous data with skewed distribution or with mean and standard deviation for variables with normal distribution. The normality of distribution of quantitative variables was tested by Kolmogorov–Smirnov test.

The Mann–Whitney test was applied for comparison of non-normally distributed variables. Categorical variables were expressed as percentages and tested by the chi-squared test.

Data were analysed by developing themes related to the specific objectives. These themes were knowledge, attitude, and practice levels. Each participant was assigned a knowledge score, an attitude score, and a practice score based on the number of correct responses for knowledge, good practice, and positive attitude.

The scores were further analysed using linear regression analysis to determine independent effects of explanatory variables or predictors of each score (knowledge, attitudes, and practices) in both univariate and multivariable analysis.

Knowledge, attitude, and practice were the primary outcome variables. Explanatory variables included: age, number of children, mother’s educational level, mother’s marital status, children’s health status, and income.

Predictors with *p* value ≤0.25 in univariate analysis were introduced in multivariable analysis. Assumptions of residuals being normally distributed were fulfilled for all scores using graphical method. Significance was predetermined at a probability value of 0.05 or less.

Resulting associations were reported as regression coefficients β and their confidence intervals (95% CI). Data analysis was conducted using SPSS 13.0 software (Chicago, IL, USA).

## Results

A total of 503 mothers were included during the study period. Ten mothers refused to respond to the all items of the questionnaire.

The socio-demographic characteristics of the included mothers are represented in Table 1. The mean age of the studied mothers was 30.3 ± 6.7; 461 (91.8%) of the mothers were non-employed. The median number of children was 2(1-3).

**Table 1:** Characteristics of the studied population

Variables	Values
	*N* = 502
Age, yrs. (mean ± SD)	30.3 ± 6.7
Age of mother at birth of the first children, yrs. (mean ± SD)	23.6 ± 5
Number of children, median[IQR]	2[1–3]
Mother’s employment status, n(%)	
Non working	387(76.9)
Working	116(23.06)
Mother’s educational level, n(%)	
None	76(15.1)
1 to 5 years	128(25.5)
6 to 12	228(45.4)
University	52(10.3)
No response	19(3.6)
Family income, n(%)	
Low	195(38.6)
Medium	109(21.6)
High	116(23.06)
No response	83(16.5)
Knowledge score, [median (IQR)]	5[4–6]
Attitude score, median [IQR]	2[1–3]
Practices score, median [IQR]	2[1–3.75]

*IQR* interquartile range, *SD* standard deviation

Regarding educational background, 140 (27.8%) of the mothers were illiterate and 129 (25.6%) of the mothers were primary-school level; 372 (74%) had medical coverage. The median knowledge score was 5(4-6).

A total of 370 (95.9%) of the mothers were unaware that teeth should be cleaned beginning at eruption. The median age of tooth-brushing inception declared by the mothers was 3.5(3-5) of the mothers studied, 253 (53.3%) believed that primary teeth are not necessary and that more care should be taken for permanent teeth, 285 (60.9%) of the mothers were aware that fluoride has a beneficial effect in prevention, and 409 (86.3%) of the mothers were unaware that medication intake during pregnancy and childhood may affect teeth development.

The main sources of information cited by mothers were family members and other mothers. Paediatricians and dentists were cited in less than 10% of cases.

Previous children’s use of dental services was reported by 230 (45.7%) of the mothers. Emergency dental care was reported by 46.9% of the mothers, and 69 (13.7%) of the mothers reported that they sought treatment for their children from a dental practitioner for a toothache.

Mothers’ own practices about dental care were statistically related to their children’s use of dental care (*p* < 0.001). Among mothers who had never had a previous dental consultation, 121 (77.1%) declared that their children had never sought a dentist’s care while 78 (41.7%) of mothers who had received dental care had sought a previous consultation for their children.

The findings of the linear regression analysis, examining predictors of knowledge, attitude, and practices score, are presented in Tables 2, 3 and 4.

**Table 2:** Linear regression analysis of predicators of attitudes scores

	Univariate analysis			Mulivariate analysis		
	β	95% CI	p	βadj	95% CI	p
Age of mother	− 0.03	(− 0.08, 0.02)	0.25	− 0.03	(− 0.10,0.03)	0.30
Age of mother at birth of the first children	−0.05	(− 0.02,-0.13)	0.02	0.009	(−0.08,0.10)	0.86
NUMBER OF CHILDERN	−0.23	(−0.52, 0.06)	0.12	0.10	(−0.21,0.43)	0.50
Mother’s Educational level						
None	1			1		
1 to 5 years	−0.22	(− 1.13, 0.67)	0.61	− 0.06	(− 1.09, 0.96)	0.89
6 to 12	0.32	(−0.30, 0.96)	0.30	0.14	(−0.63, 0.92)	0.71
University	1.38	(0.05,2.71)	0.04	1.7	(0.26,3.14)	0.02
Mother’s employment status, working^a^	1.42	(0.34,2.50)	0.01	1.73	(0.68,2.79)	0.002
Children health status, chronic illness^b^	0.68	(−0.17,1.54)	0.11	−0.20	(−1.11,0.70)	0.65
Family income						
Low	1			1		
Medium	1.46	(0.73,2.19)	< 0.001	1.47	(0.64,2.30)	0.001
High	0.82	(−0.67,2.32)	0.27	0.62	(− 0.96,2.21)	0.43

*1* reference category, *β* unadjasted linear regression coefficient, *βadj* adjasted linear regression coefficient, *CI* confidence interval

^a^non working was reference category

^b^good global health was reference category

**Table 3:** Linear regression analysis of predicators of knowledge score

	Univariate analysis			Mulivariate analysis		
	β	95% CI	p	βadj	95% CI	p
Age of mother	−0.03	(− 0.08,0.02)	0.25	0.05	(0.03,0.08)	< 0.0001
Age of mother at birth of the first children	- 0.05	(−0.02,-0.13)	0.02	−0.01	(−0.04,0.01)	0.39
NUMBER OF CHILDERN	−0.23	(−0.52,0.06)	0.12	0.09	(−0.20,0.42)	0.50
Mother’s Educational level						
None	1			1		
1 to 5 years	−1.04	(−1.38,-0.70)	< 0.001	0.49	(0.07,0.92)	0.02
6 to 12	−0.74	(−1.13,-0.37)	< 0.001	1.18	(0.79,1.58)	< 0.001
University	1.11	(0.56,1.65)	< 0.001	2.07	(1.43,2.71)	< 0.001
Mother’s employment status, working^a^	1.42	(0.34,2.50)	0.01	1.70	(0.69,2.8)	0.003
Children health status, chronic illness^b^	−0.07	(−0.45,0.30)	0.70			
Family income						
Low	1			1		
Medium	0.60	(0.22, 0.99)	0.002	0.38	(0.01,0.76)	0.04
High	- 1.20	(−0.32,-2.09)	0.007	0.45	(− 0.43,1.33)	0.31

*1* reference category, *β* unadjasted linear regression coefficient, *βadj* adjasted linear regression coefficient, *CI* confidence interval

^a^non working was reference category

^b^good global health was reference category

**Table 4:** Linear regression analysis of predicators of practices score

	Univariate analysis			Mulivariate analysis		
	β	95% CI	p	βadj	95% CI	p
Age of mother	−0.006	(− 0.04,0.03)	0.74			
Age of mother at birth of the first children	0.02	(−0.02,0.07)	0.36			
Mother’s Educationnel level						
None	−1.04	(−1.38, −0.70)	< 0.001	− 0.72	(−1.41,-0.50)	< 0.001
1 to 5 years	− 0.74	(−1.13,-0.37)	< 0.001	− 0.02	(− 0.56,-0.82)	0.02
6 to 12	1.11	(0.56, 1.65)	< 0.001	−1.18	(− 0.78,-0.33)	< 0.001
University	1				1	
Mother’s employment status, working^a^	−0.34	(−1.89, 1.20)	0.65			
Children health status, chronic illness^b^	0.87	(0.30,1.45)	0.003	0.84	(0.25,1.43)	0.005
Family income						
Low	1			1		
Medium	0.45	(−0.15, 1.06)	0.14	0.32	(− 0.29, 0.93)	0.30
High	0.94	(−0.18, 2.06)	0.10	1.12	(−0.04, 2.29)	0.06

*1* reference category, *β* unadjasted linear regression coefficient, *βadj* adjasted linear regression coefficient, *CI* confidence interval

^a^non working was reference category

^b^good global health was reference category

The adjusted linear regression model showed that the knowledge score was positively related to mother’s age (β = 0.05; *p* < 0.001), education level, and median income (β = 0.38; *p* = 0.04).

Significant predictors of oral-health-related practices were mother’s education and children’s health status. The predictors of oral-health-related attitudes were income, mother’s employment status, and education level.

## Discussion

The findings of the present study indicated a low level of knowledge and unfavourable attitudes and practices related to oral health. Socio-economic factors were the main predictors of KAP scores.

Parents’ knowledge and attitudes predicted a range of negative oral health practices. Less knowledge of parenting strategies to promote healthy teeth may be a predictor of more unhealthy teeth [10–15].

Regular use of fluoride to control dental caries was reported in many studies [16, 17, 20, 21]. However, fluoride use in the present study was not widespread. These results may be explained by low knowledge of fluoride’s role in prevention of dental caries. Studies in various countries showed that low adherence to preventive measures was associated with high prevalence of dental caries [11, 22–24].

The development of positive and negative patterns in children (oral health behaviour, hygiene attitudes, sugar consumption) are a function of parental feeding practices [25, 26]. The present study revealed a lower rate of adequate oral-health-related attitudes and practices among both mothers and children.

Parents constitute an important social model in delivering health skills to their children [26]. Many studies have shown that tooth-brushing behaviour established during infancy is often maintained during early childhood, adolescence, and adulthood [23, 27].

The family, and particularly mothers, provide the child’s proximate home environment [25, 26]. Being unaware of or neglecting the importance of oral health and common preventive measures against dental caries and periodontal diseases are important inhibiting factors to achieving an acceptable level of children’s oral health [16, 17, 28].

Thus, lack of awareness and corollary attitudes and practices likely contribute to a high prevalence of common oral health diseases (dental caries and periodontitis), and the associated costs impose a substantial burden on societies [16, 17, 28]. As role models, parents can encourage oral health preventive measures by regularly encouraging children to brush and brushing themselves [26]. Parents should also set eating examples by encouraging less use of sweetened beverages (particularly sweetened milk) [29–31].

As the results of this study revealed, there are still a considerable number of mothers who prefer other options to taking the child to the dentist, even when child has an emergency need. To some extent, this may predispose the child to greater dental health complications and impact nutritional status. A possible reason for this low prevalence may be attributable to economic limitations. Studies have shown lower rates of dental care use among low socio-economic groups [32, 33].

The present study showed that the level of health-related knowledge increased with years of schooling. Mothers with higher education are thought to have better opportunity to obtain information about childcare than mothers with lower education levels [34, 35]. Mothers with less education may not have basic knowledge about the impacts of potential risk factors on the occurrence of preventable oral diseases [34, 35]. Therefore, health education and promotion are warranted to be developed among mothers with low educational background [36–38].

Dental caries and periodontal diseases are common and frequently occurring oral diseases among the Moroccan population [16]. To control these diseases, it is not enough to focus on the surgical treatment, as subjective factors such health literacy and self-management efficacy also have great impact [38].

Within families, social and cultural norms are known to shape many attitudes towards health [39, 40]. In the present study, the mothers followed the advice of their circle of mothers and those in their community. This attitude reaffirms traditional values independently of their relevance, and highlights the role of social network transmission of health behaviours [40–42].

In the context of the present study, 140 (27.8%) of the 502 mothers included were illiterate. To a certain extent, therefore, greater attention to barriers to seeking and using medical advice should be considered [42].

Health- related behaviour is affected by different aspects of knowledge, attitude, and practices [25, 26, 38]. This highlights the role of educational interventions affecting the health condition of one individual that could also affect the health condition of others in his/her environment [25, 26, 38, 40, 42].

Mother’s educational level and income were significantly inversely related to children’s oral health practices and attitudes. Greater income among families with working mothers and greater educational level could explain findings results regarding attitude scores. These socio-economic predictors are associated with higher levels of oral diseases [11, 43, 44].

The level of family income was identified as significant predictor of KAP scores among the studied population. Low socio-economic status, poor oral health knowledge and attitudes, and poor oral health behaviours are risk factors for dental caries and periodontal diseases [28]. For this, socioeconomic (SES) inequality remains an important focus in health research because economic inequality is associated with a variety of negative health outcomes [45]. Hobdell MH et al. [46] stated that socio-economic variables account for approximately 50% of the differences in the prevalence of dental caries.

The influence of SES on health outcomes may vary depending on the indicator used [47]. The present study used two indicators of SES: mother’s education level and income. These indicators were chosen because it is assumed that they are associated with material factors, psychological factors, and health behaviour [48].

The present study found that having at least one child with a health concern is associated with increased level of positive attitude. This result may be explained by the fact that having a child with a health concern may lead to better parental awareness of and attention to overall health [49].

Preventing chronic oral diseases such as dental caries and periodontitis is a result of multiple intervention strategies, including biological, environmental, and behavioural factors [1, 11, 26–28, 43, 50].

Even if immense efforts are made to control caries prevalence, it should be borne in mind that monitoring and evaluation of knowledge, attitudes, and practices in a community have a major role in creating sustainable control interventions [2, 28].

When moving from the clinical to population level, characteristics of the target population for prevention strategies should be taken into account [36, 37]. A dynamic solution to this complex problem will introduce challenges for the use of knowledge of preventive measures and its dissemination [50] among groups at high risk for oral diseases.

From this point of view, early and accurate selection of high-risk groups for prevention and intervention is of great importance. Coordinating action across health services may be a useful tool. Mother and Child units seem to be one of the most important targeted units for dissemination of primary prevention knowledge. Because vaccination programs are promoted in these units, every mother can access important knowledge and not be disadvantaged from attaining it because of class, socioeconomic status, or determined circumstance. Educational protocols need to be established to advise all mothers attending these units regarding many aspects of health, including oral health. Mothers can then receive counselling for their children from a professional who is taught to have codified knowledge. However, ensuring optimal dissemination of the best knowledge on oral health promotion presents an ongoing challenge and requires improved awareness among health professionals working in the MCUs.

Some limitations should be addressed for the present study. Its cross-sectional design limits the ability to conclude a causal inference. However, the predictors included (mother’s education, income, and employment status) are unlikely to change over time.

Another limitation that should be taken into account is the high rate of mothers in the low and medium income category. This could influence the results, and a repetition of the study design should take into account this limitation.

However, this study also has some strengths. Firstly, the findings add data to the limited literature on oral-health-related KAPs among mothers from countries with a high oral disease burden.

Moreover, these findings have important implications for future research. Acknowledgement of cultural practices that reflect inappropriate attitudes and practices should be addressed for more contextual preventive measures. Research to improve public perceptions, as well as the perceptions of mothers specifically, regarding oral health may lead to greater improvement of oral health indicators.

Better understanding of the importance of dental health in daily life and better oral hygiene could enhance oral health indicators and reduce the burden of dental caries among specific groups.

Health professionals, together with health workers, should perform counselling activities to address the risk factors associated with the disease. Mothers attending MCUs are a key target population for education regarding all aspects of health. In this context, health education should be planned to increase knowledge and minimise misconceptions of the mothers as well as the community as a whole.

## Conclusion

The results of this study indicated that socio-economic factors such as mother’s educational level and income were predictors for oral health practices and attitudes. These findings call for primary care services, particularly maternal and child health services, to increase oral health knowledge. Oral health education and promotion workshops should be organised in maternal and child health units to educate and inform mothers regarding dental caries prevention measures.

## Additional file

Additional file 1: Knowledge, attitudes and self-reported practices toward children oral health among mother’s attending maternal and child’s units, Salé, Morocco. (DOCX 18 kb)
