# Supplementary material for: Knowledge, attitudes and self-reported practices toward children oral health among mother’s attending maternal and child’s units, Salé, Morocco
Source: BMC Public Health. 2018 May 11;18:618. doi: 10.1186/s12889-018-5542-2 (PMC5948847; doi:10.1186/s12889-018-5542-2)
Supplement: Supplementary file 1 — Knowledge, attitudes and self-reported practices toward children oral health among mother’s attending maternal and child’s units, Salé, Morocco. (DOCX 18 kb) [file 12889_2018_5542_MOESM1_ESM.docx]

**Socio- demographic factors**

Maternal and Child health unit:___________

Mother´s age.: ___________

Age of mother at birth of the first children:

Mother´s educational level: 🞏None 🞏 1 to 5 years 🞏6 to 12 years 🞏University

Family income  : 🞎 low 🞎medium 🞎High

Mother’s employment status: 🞏 Non working 🞏 working

Medical coverage: 🞏 Yes 🞏 No

| Child 1 | Age  ____ years | Gender:  🞏 M  🞏 F | Child health status  🞏 Chronic illness 🞏Good health status | Age at beginning of tooth brushing: ____ years |
| --- | --- | --- | --- | --- |
| Child 2 | Age | Gender:  🞏 M  🞏 F | Child health status  🞏 Chronic illness 🞏Good health status | Age at beginning of tooth brushing: ____ years |
| Child 3 | Age | Gender:  🞏 M  🞏 F | Child health status  🞏 Chronic illness 🞏Good health status | Age at beginning of tooth brushing: ____ years |
| Child 4 | Age | Gender:  🞏 M  🞏 F | Child health status  🞏 Chronic illness 🞏Good health status | Age at beginning of tooth brushing: ____ years |
| Child 5 | Age | Gender:  🞏 M  🞏 F | Child health status  🞏 Chronic illness 🞏Good health status | Age at beginning of tooth brushing: ____ years |
| Child 6 | Age | Gender:  🞏 M  🞏 F | Child health status  🞏 Chronic illness 🞏Good health status | Age at beginning of tooth brushing: ____ years |

| **Mother´s health status**  🞏 Chronic illness  🞏 Good health status  **Practices**  **-** Do you brush your teeth regularly?  🞏 yes 🞏 no  **- Y**our child brush his teeth?  🞏 yes 🞏 no  -If yes, teeth are brushed regularly?  🞏 yes 🞏 no  - Which brushing motion you use to brush your teeth  🞏 vertical 🞏 horizontal  🞏 another to specify  -If yes, are teeth brushed using a toothpaste containing fluoride?  🞏 Yes 🞏 No 🞏 Do not Know  - Did you give sweet milk in the bottle to your child?  🞏 Yes  🞏 No  -Frequency of tooth brushing: _______/ day  **-** Duration of teeth brushing ….. Min  - Your child use a personal toothpaste or familial toothpaste? 🞏 Yes 🞏 No  - Do you use the same spoon as your child?  🞏 Yes 🞏 No  - Have you ever visit a dentist?  🞏 Yes 🞏 No  - If yes, what was the reasons:  🞏Emergency need  🞏 Check up  🞏Aesthetic concern  🞏 Another reason, specify  - Have your child ever visit a dentist?  🞏 Yes 🞏 No  - If yes, what was the reasons:  🞏Emergency need  🞏 Check up  🞏Aesthetic concern  🞏 Another reason, specify | **attitudes**  - What was your child´s attitude when brushing his teeth when having less than 2 years old?  🞏 Cooperative  🞏 uncooperative  🞏 I Do not remember  - Did you assist your children brushing teeth when having less than 2 years old?  🞏 Yes 🞏 No  -When your children complains of dental pain, what do you do?  🞏 you consult a dentist  🞏 you give him an analgesic  🞏 you give him a medicinal plant  🞏 other proposition: …………  - Have you used to soothe your child by soaking his pacifier in honey  🞏 Yes  🞏 No  - Have you ever wipe your child´s pacifier with your saliva?  🞏 Yes  🞏 No  - Who advices you regarding your child´s feeding and health concern:  🞏 No one  🞏 your pediatricians  🞏 other health workers to precise  🞏 your family  🞏 other mother´s  🞏 other persons to specify |
| --- | --- |
|  |  |

| **Knowledge:**  - What is the recommend age to start tooth brushing: ____ years  -Should teeth be brushed using a fluoridated toothpaste? 🞏 Yes 🞏 No  🞏 Do not Know  -What is the recommended dose of fluoride to choose for your children?  🞏 ____ 🞏 Do not Know  -Should teeth been brushed regularly?  🞏 yes 🞏 no  🞏 Do not Know  -What is the recommended frequency of teeth brushing  🞏 ____ 🞏 Do not Know  -General body health has a relationship with oral health  🞏 yes 🞏 no 🞏 Do not Know  -Dental caries complications related to primary teeth may affect permanent teeth  🞏 yes 🞏 no 🞏 Do not Know | - sugars are an important factor for dental caries development  🞏 yes 🞏 no 🞏 Do not Know  - oral bacteria may be transmitted from mother to child by using the same spoon:  🞏 yes 🞏 no 🞏 Do not Know  - What is the importance of oral health compared to general health:  🞏 the same importance  🞏 more important  🞏 less important  🞏 Do not Know |
| --- | --- |
